# Hot−yet−suppressed under PD−1 blockade: an RMP–NRF2–PD−L1 axis associated with a reduced proportional response in hepatocellular carcinoma

**DOI:** 10.3389/fimmu.2026.1737569

**Published:** 2026-02-05

**Authors:** Mingzhu Zuo, Haiqiang Li, Na Chen, Zengjun Guo, Zhenghua Wan

**Affiliations:** 1 Department of Medical Oncology, The First Affiliated Hospital of Xi’an Jiaotong University, Xi’an, China; 2 School of Pharmacy, Xi’an Jiaotong University, Xi’an, China; 3 Department of Oral and Maxillofacial Surgery, School of Stomatology, The Fourth Military Medical University, Xi’an, China; 4 Department of Rehabilitation Medicine, The First Afffliated Hospital of Xi’an Jiaotong University, Xi’an, China

**Keywords:** adaptive immune resistance, hepatocellular carcinoma, Nrf2, PD-L1, RMP

## Abstract

Immune checkpoint blockade (ICB) provides therapeutic benefits to a subset of patients with hepatocellular carcinoma (HCC); however, reliable predictors of treatment efficacy remain scarce. This study investigates whether RPB5-mediating protein (RMP) facilitates the alignment of redox adaptation with immune checkpoint regulation, thereby influencing the extent of therapeutic benefit under programmed cell death protein 1 (PD-1) blockade. In Hepa1–6 and Hep3B cell lines, enforced expression of RMP resulted in elevated levels of NRF2 and PD-L1 proteins, alongside enhanced clonogenic growth and short-term migratory capacity. In a subcutaneous Hepa1–6 tumor model, RMP-overexpressing tumors exhibited accelerated growth and a distinct immunohistochemical profile characterized by increased levels of RMP, NRF2, PD-L1, Ki-67 and HO-1, indicative of a proliferative and redox-adapted state. Upon administration of anti-PD-1 therapy, both experimental cohorts demonstrated tumor regression; however, the RMP-overexpressing cohort exhibited a proportionally reduced inhibition compared to controls, despite experiencing greater absolute tumor shrinkage from a higher baseline. This suggests a limited response amplitude within the RMP/NRF2-high context. Post-therapy tissues from the overexpression cohort exhibited elevated levels of RMP, NRF2, HO-1, and PD-L1, alongside an immune microenvironment characterized by an increased presence of CD3/CD8 cells and a decreased presence of CD4/CD25 cells. This pattern is indicative of an inflamed yet suppressed state of adaptive immune resistance. Collectively, these observations support a model wherein continuous RMP–NRF2–HO-1 activity and persistent PD-L1 expression exert inhibitory pressure, even as PD-1 blockade facilitates cytotoxic T-cell infiltration. This dynamic accounts for the relatively lower inhibition observed in the overexpression context. The combined RMP/NRF2/PD-L1 signature proposes a mechanistically informed biomarker framework and suggests the potential for rational therapeutic combinations that pair PD-1 blockade with modulation of the redox pathway in HCC.

## 1 Introduction

Immune checkpoint blockade (ICB) has significantly transformed the therapeutic landscape of hepatocellular carcinoma (HCC). First-line treatment regimens, including the combination of atezolizumab and bevacizumab as well as the STRIDE regimen, have demonstrated improvements in overall survival in phase III clinical trials (1–4). However, the clinical benefits observed remain heterogeneous among patients and across different disease contexts. This variability highlights the limitations inherent in single-marker strategies for HCC; for instance, programmed death-ligand 1 (PD-L1) alone exhibits inconsistent predictive value. Consequently, there is an increasing demand for integrative biomarkers and context-aware stratification approaches (5–10). Conceptually, the outcomes associated with PD-1/PD-L1 blockade are reflective of the dynamic interplay between an inflamed, T-cell–infiltrated tumor microenvironment and adaptive inhibitory mechanisms, such as interferon-gamma–inducible PD-L1. This hot-yet-suppressed state is frequently linked to responsiveness, albeit with a variable magnitude of benefit (11–15).

At the molecular level, the KEAP1-NRF2 signaling pathway serves as a pivotal regulator of redox adaptation and plays a significant role in modulating anti-tumor immunity (16, 17). Across various tumor types, elevated NRF2 activity and alterations in the KEAP1 pathway have been associated with reduced efficacy of PD-1-based therapies and the activation of immunoregulatory programs that can suppress effector T-cell function (18–22). These immunoregulatory programs may manifest as immune exclusion in certain contexts, while in others, they may present as an inflamed yet suppressed phenotype indicative of adaptive resistance under checkpoint blockade (11, 23–25). Further downstream, heme oxygenase-1 (HO-1), a well-established NRF2 target, contributes to the immunoregulatory environment and has been implicated in myeloid-driven suppression, thereby providing a mechanistic pathway through which inflamed tumors can maintain functional restraint (26–30). Upstream of NRF2, the RPB5-mediating protein (RMP, also known as URI1) has been identified as a coordinator of stress responses; in models of biliary and liver cancer, RMP can interact with KEAP1 to stabilize NRF2, thereby promoting antioxidant responses, cellular survival, and therapy tolerance (31–33). Despite these converging threads, in hepatoma the integrated relationships among RMP, NRF2, and PD−L1—and how this composite state relates to the proportional benefit from PD−1 blockade—remain insufficiently defined in a disease where validated predictive biomarkers are still lacking.

**Figure 1:**
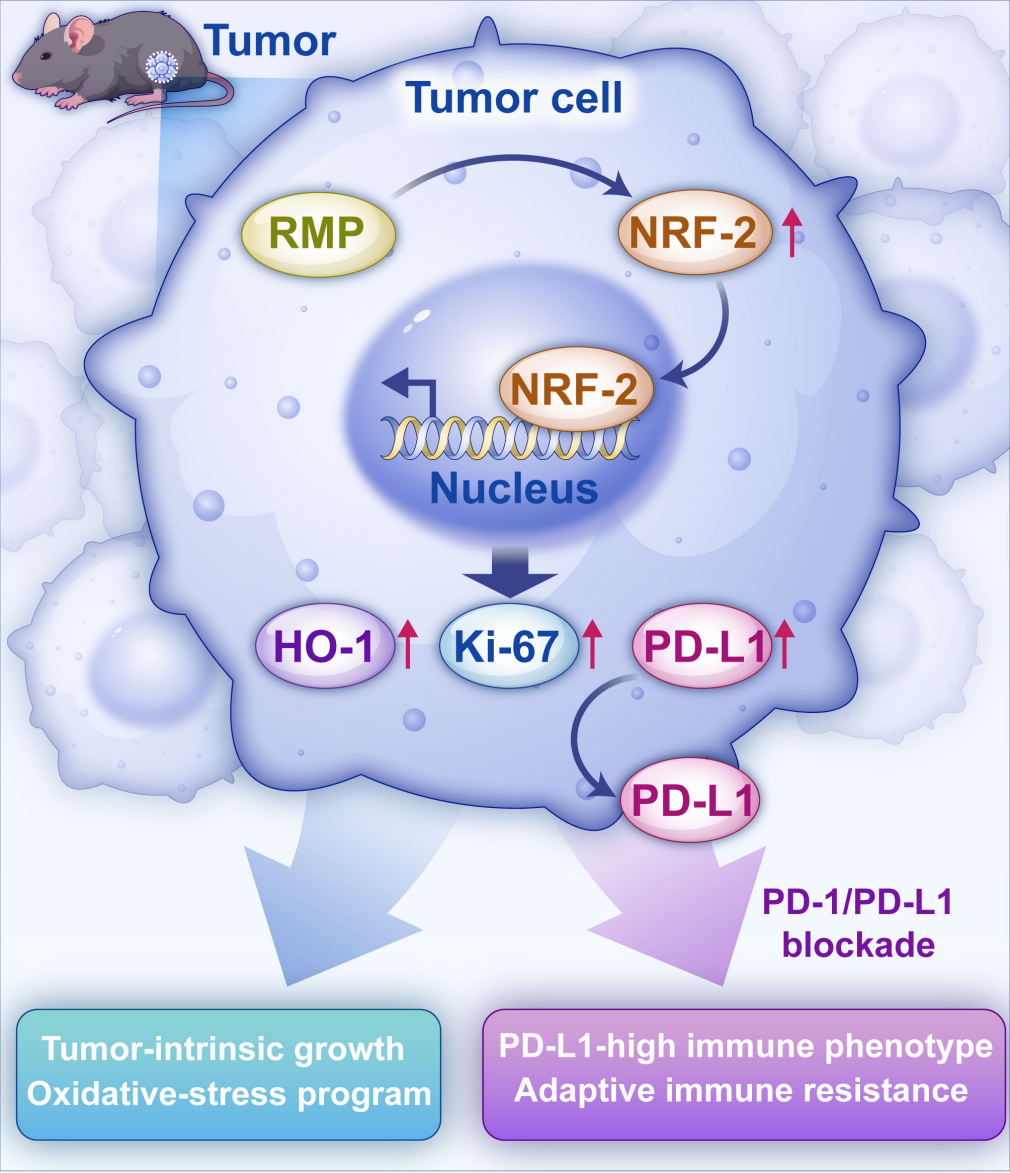
RMP–NRF2 axis links tumor-intrinsic fitness with PD-L1–mediated T-cell inhibition and shapes response to PD-1/PD-L1 blockade. Schematic of the proposed model in hepatoma: RMP upregulation stabilizes/activates NRF2 and promotes its nuclear translocation, which increases transcriptional programs associated with proliferation (Ki-67) and redox adaptation (HO-1) and concomitantly upregulates PD-L1. Elevated PD-L1 restrains T-cell activity (“hot yet suppressed” contexture). PD-1/PD-L1 blockade relieves T-cell inhibition and enhances antitumor immunity. RMP, RP-associated protein (URI1); NRF2, nuclear factor erythroid 2–related factor 2; PD-L1, programmed death-ligand 1; PD-1, programmed cell death protein 1; HO-1, heme oxygenase-1.

In this study, we investigate a mechanistic hypothesis rooted in the proposed framework. We hypothesized that enforced expression of RMP would synchronize tumor-intrinsic growth and oxidative stress pathways with a PD-L1-high immune phenotype, thereby creating conditions conducive to adaptive immune resistance during PD-1 blockade. Specifically, under PD-1 inhibition, we expected an increase in cytotoxic T-cell infiltration indicative of pathway activation, persistence of the RMP-NRF2-HO-1 axis, and sustained PD-L1 expression. This would maintain inhibitory pressure and reduce the relative percentage of inhibition in RMP-overexpressing tumors compared to controls, even if there is absolute tumor shrinkage. (**Figure 1**) To test this hypothesis, we engineered Hepa1–6 and Hep3B cells to overexpress RMP, conducted immunoblotting to profile RMP/NRF2/PD-L1, assessed clonogenic growth and migration, and examined *in vivo* growth and tissue characteristics using immunohistochemistry and dual immunofluorescence, including markers such as Ki-67 and HO-1. In a subcutaneous model, we compared tumor control under anti–PD-1 treatment across different genotypes. This approach provides a composite biomarker framework and informs rational PD-1–based, redox-targeted therapeutic combinations in HCC.

## 2 Results

### 2.1 RMP overexpression establishes an RMP–NRF2–PD−L1 axis in hepatocellular carcinoma cells

**Figure 2:**
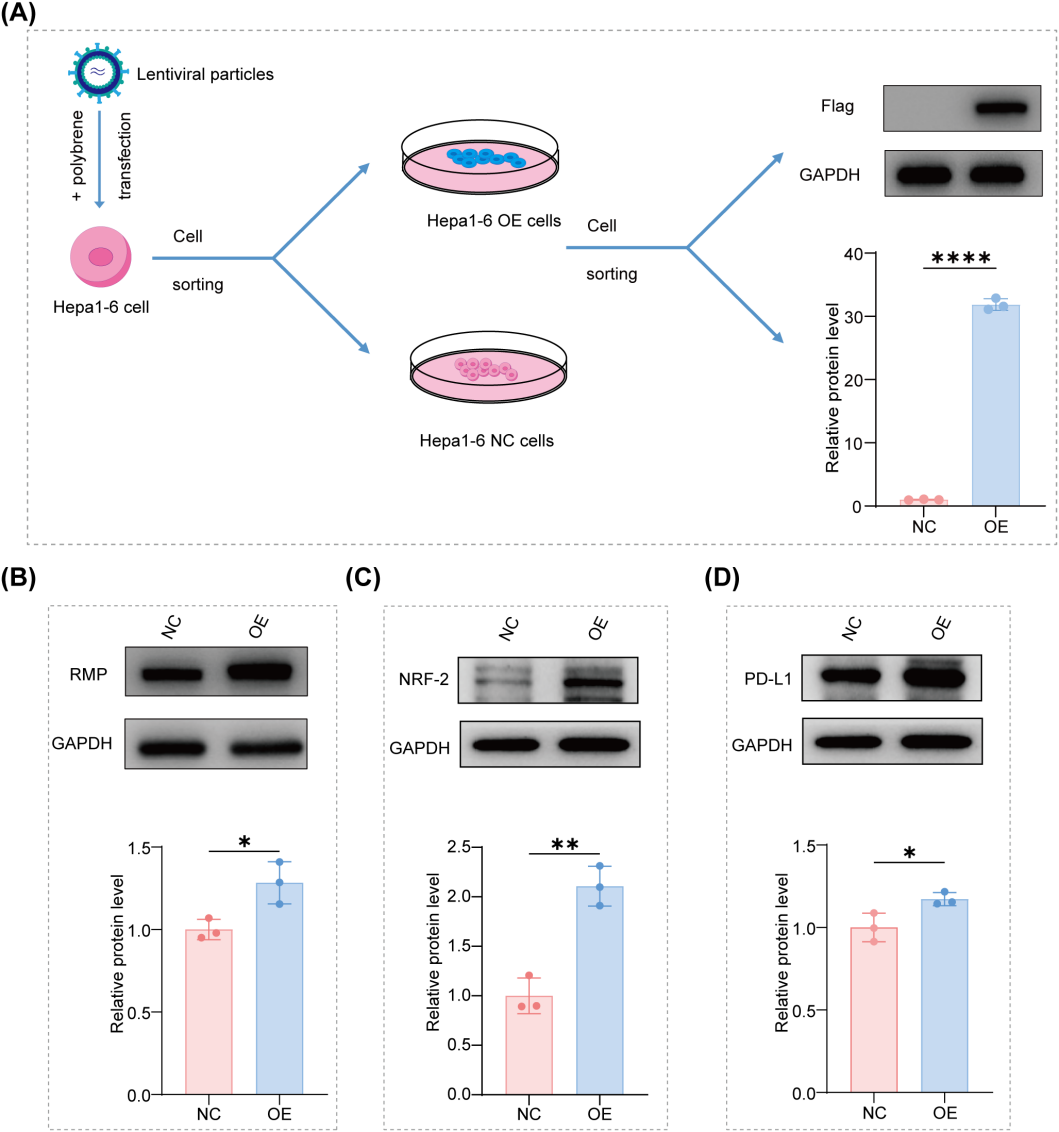
RMP overexpression increases NRF2 and PD−L1 protein levels in Hepa1−6 cells. **(A)** Schematic of lentiviral transduction (with polybrene) and sorting to establish RMP-overexpression (OE) and negative control (NC) Hepa1–6 cells. Representative Western blot validating RMP overexpression using a Flag tag. **(B**–**D)** Representative blots and densitometric quantification of RMP **(B)**, NRF2 **(C)**, and PD-L1 **(D)**. Data are presented as mean ± SD, n=3 independent experiments and comparisons were performed with Student’s t-test; *p<0.05, **p<0.01, ****p<0.0001.

To elucidate the impact of enforced RMP expression on canonical redox and immune checkpoint pathways in hepatoma, we established stable RMP-overexpressing (OE) cell lines in murine Hepa1–6 and human Hep3B cells via lentiviral transduction followed by antibiotic selection. The robust overexpression of epitope-tagged RMP was initially confirmed through immunoblotting, compared to negative-control (NC) lines generated concurrently. (**Figure 2A**) Densitometric analysis, normalized to GAPDH and then to the NC mean, consistently demonstrated a significant increase in RMP protein levels across three independent experiments for each cell line, thereby validating the intended perturbation (**Figure 2B**). In the same lysates, we examined NRF2 and PD-L1, two critical effectors associated with oxidative stress adaptation and adaptive immune resistance, respectively. In Hepa1–6 cell lines, NRF2 protein levels were elevated relative to NC, and PD-L1 levels were also increased, resulting in a profile characterized by elevated RMP, NRF2, and PD-L1 at steady state. This pattern was consistently reproducible across replicates and achieved statistical significance, as denoted by the asterisk scheme in the plots (**Figures 2C, D**). The data suggest that the elevation of RMP is adequate to stabilize or enhance NRF2 and to increase PD-L1 levels in hepatoma cells, thereby establishing a cell-intrinsic RMP-NRF2-PD-L1 axis at the protein level. Importantly, flow cytometry further confirmed that RMP overexpression increased the fraction of PD−L1–positive cells at the cell surface, supporting the immunologically relevant, membrane-associated PD−L1 elevation downstream of the RMP–NRF2 program (**Supplementary Figures S1A, B**). To investigate the dependence of PD-L1 regulation on NRF2 within the RMP-OE context more thoroughly, Hepa1–6 RMP-OE cells were treated with the NRF2-suppressive agent, brusatol. Immunoblot analysis demonstrated a reduction in NRF2 protein levels, which was associated with a corresponding decrease in PD-L1 expression compared to the control group (**Supplementary Figures S2A, B**). In addition, exposure of Hepa1−6 RMP−OE cells to H_2_O_2_ as an oxidative challenge further increased NRF2 protein levels and was accompanied by a parallel upregulation of PD−L1 (**Supplementary Figures S3A, B**), supporting a redox-responsive association between NRF2 activation and PD−L1 expression in the RMP-high context.

Notably, a comparable trend was observed in human Hep3B cells, wherein the overexpression of RMP was associated with elevated levels of NRF2 and PD-L1 proteins in comparison to the negative control (**Supplementary Figures S4A, B**). In alignment with the observed coupling at the protein level, an analysis of the TCGA-LIHC cohort further corroborates a positive association between URI1 (RMP) and NFE2L2 (NRF2), as well as PD-L1 (CD274). (**Supplementary Figures S5A, B**) This association occurs with minor alterations in baseline immune infiltration, highlighting the clinical significance of the RMP–NRF2–PD-L1 state and providing a rationale for our subsequent functional evaluation of RMP-driven tumor cell fitness.

### 2.2 RMP overexpression promotes clonogenic growth and short−term migration *in vitro*

**Figure 3:**
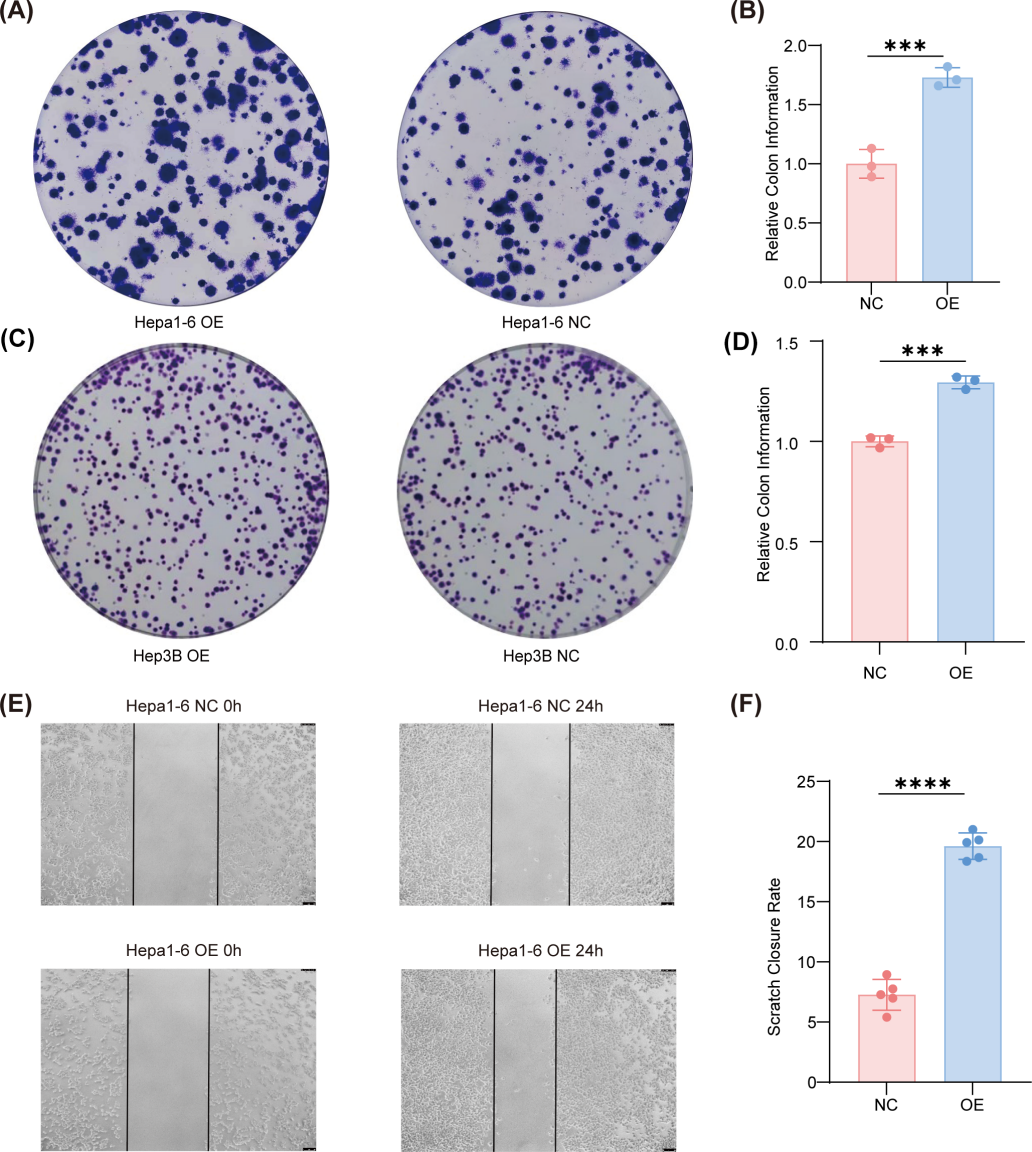
RMP overexpression promotes clonogenic growth and migration of HCC cells. **(A–D)** Crystal-violet colony-formation assay. Representative plates and quantification for Hepa1-6 **(A, B)** and Hep3B **(C, D)** cells under overexpression (OE) or negative control (NC) conditions (normalized to NC). OE significantly increases colony formation in both lines (n=3). **(E, F)** Wound-healing assay of Hepa1-6. Representative images at 0 h and 24 h **(E)** and quantification of scratch-closure rate at 24 h **(F)** (n=5). The data were presented as mean ± SD. and comparisons were performed with Student’s t-test; ***p < 0.001, ****p<0.0001.

Building on the immunoblot evidence supporting an RMP–NRF2–PD-L1 axis, we proceeded to quantitatively assess the impact of RMP overexpression on colony-forming potential and two-dimensional migratory behavior. In the crystal-violet colony formation assay, Hepa1–6 and Hep3B overexpression (OE) lines were seeded at low density and allowed to form colonies over a 10-day period under identical culture conditions. Following fixation and staining, colonies were enumerated and normalized to the negative control (NC) group for each cell line. Across three independent experiments, OE cells exhibited a significant increase in colony numbers. Qualitative inspection of representative plates indicated a trend towards larger colony sizes, particularly in Hepa1-6 (**Figures 3A–D**).

We evaluated the migratory capacity using a scratch-wound assay on confluent Hepa1–6 monolayers. Immediately following the creation of a linear wound, images were captured at the 0-hour mark, and wound closure was quantified after 24 hours under standard serum conditions. RMP-OE monolayers demonstrated accelerated wound closure compared to NC, as indicated by a higher percentage of gap reduction over the 24-hour period (**Figures 3E, F**). Although wound-healing assays can be affected by cell proliferation, the relatively short duration of the experiment and the use of matched conditions help to mitigate this confounding factor. Furthermore, the concurrent increase in clonogenicity suggests that RMP likely influences both proliferation-associated and motility-associated characteristics. Future investigations employing transwell migration or invasion assays and live-cell tracking could more precisely delineate these components. However, within the context of our study, the combined enhancements in clonogenicity and scratch-wound closure support a role for RMP in promoting cellular fitness.

Together, these results suggest a coordinated rewiring in which RMP overexpression simultaneously pushes hepatoma cells toward a redox−competent, immune−checkpoint−high state and toward a growth−and−motility−enhanced phenotype. The biological plausibility of this coupling is supported by prior observations that RMP, also known as URI1, can engage KEAP1 via acidic motifs to limit NRF2 degradation, thereby favoring NRF2−dependent transcriptional programs; in turn, NRF2 activity has been associated with PD−L1 regulation in multiple tumor contexts (31, 34, 35).

### 2.3 RMP overexpression accelerates tumor growth and imprints a proliferative–oxidative, PD−L1−high IHC signature *in vivo*

**Figure 4:**
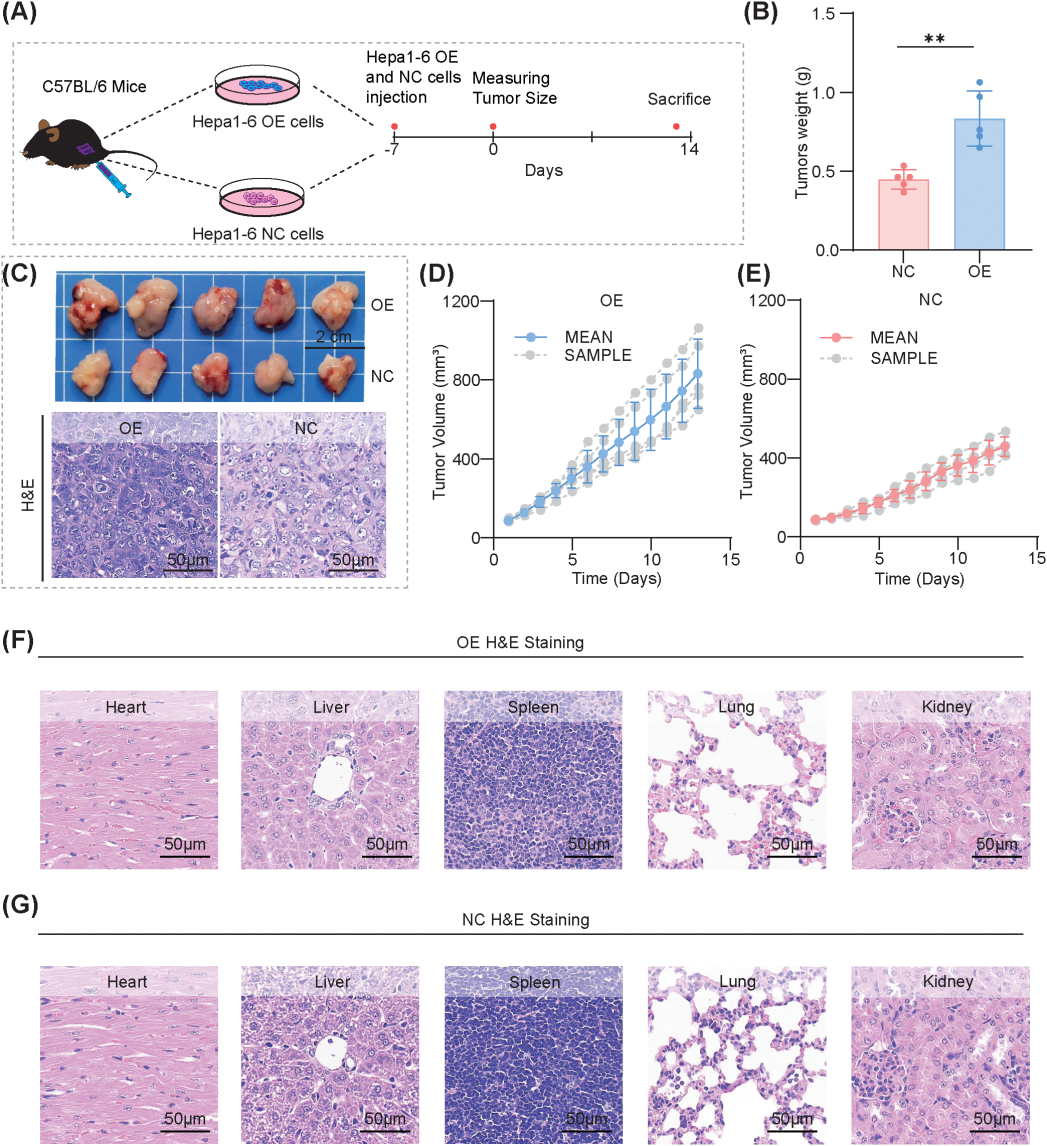
RMP overexpression accelerates subcutaneous tumor growth *in vivo* without overt histopathological abnormalities in major organs. **(A)** Schematic of the subcutaneous tumor model: Hepa1–6 RMP-overexpression (OE) and negative-control (NC) cells were injected into C57BL/6 mice, tumor volumes were measured serially and mice were sacrificed at endpoint. **(B)** Endpoint tumor weights, showing a significant increase in the OE group. **(C)** Representative images of excised tumors (scale bars: 2 cm) and H&E staining of tumor sections (scale bars: 50 μm). **(D, E)** Longitudinal tumor-growth curves for OE **(D)** and NC **(E)** cohorts with individual mice (gray) and mean ± SD. **(F, G)** H&E staining of heart, liver, spleen, lung, and kidney from OE **(F)** and NC **(G)** mice, showing no obvious histopathological abnormalities (scale bars, 50 μm). The data were presented as mean ± SD (n=5). and comparisons were performed with Student’s t-test; **p < 0.01.

Subsequently, we investigated the impact of RMP overexpression on tumor behavior *in vivo* utilizing a subcutaneous Hepa1−6 model in immunocompetent mice. An equal number of overexpression (OE) and negative control (NC) cells were implanted into the flanks of female C57BL/6 mice (n=5 per group), and tumor growth was monitored longitudinally until predetermined endpoints were reached. (**Figure 4A**) Compared to the NC group, tumors in the OE group demonstrated accelerated growth, as evidenced by increased serial volumes and higher endpoint tumor weights (**Figures 4B, D, E**). Representative gross images and hematoxylin and eosin (H&E) stained sections displayed typical hepatoma morphology without qualitative architectural differences beyond size, indicating that RMP overexpression does not significantly alter the histological phenotype in this context (**Figure 4C**). Body weight trajectories did not significantly differ between the groups (**Supplementary Figure S6**), and H&E surveys of organs revealed no overt histopathological changes in the heart, liver, spleen, lung, or kidney under baseline conditions (**Figures 4F, G**), suggesting that the model did not induce systemic toxicity.

**Figure 5:**
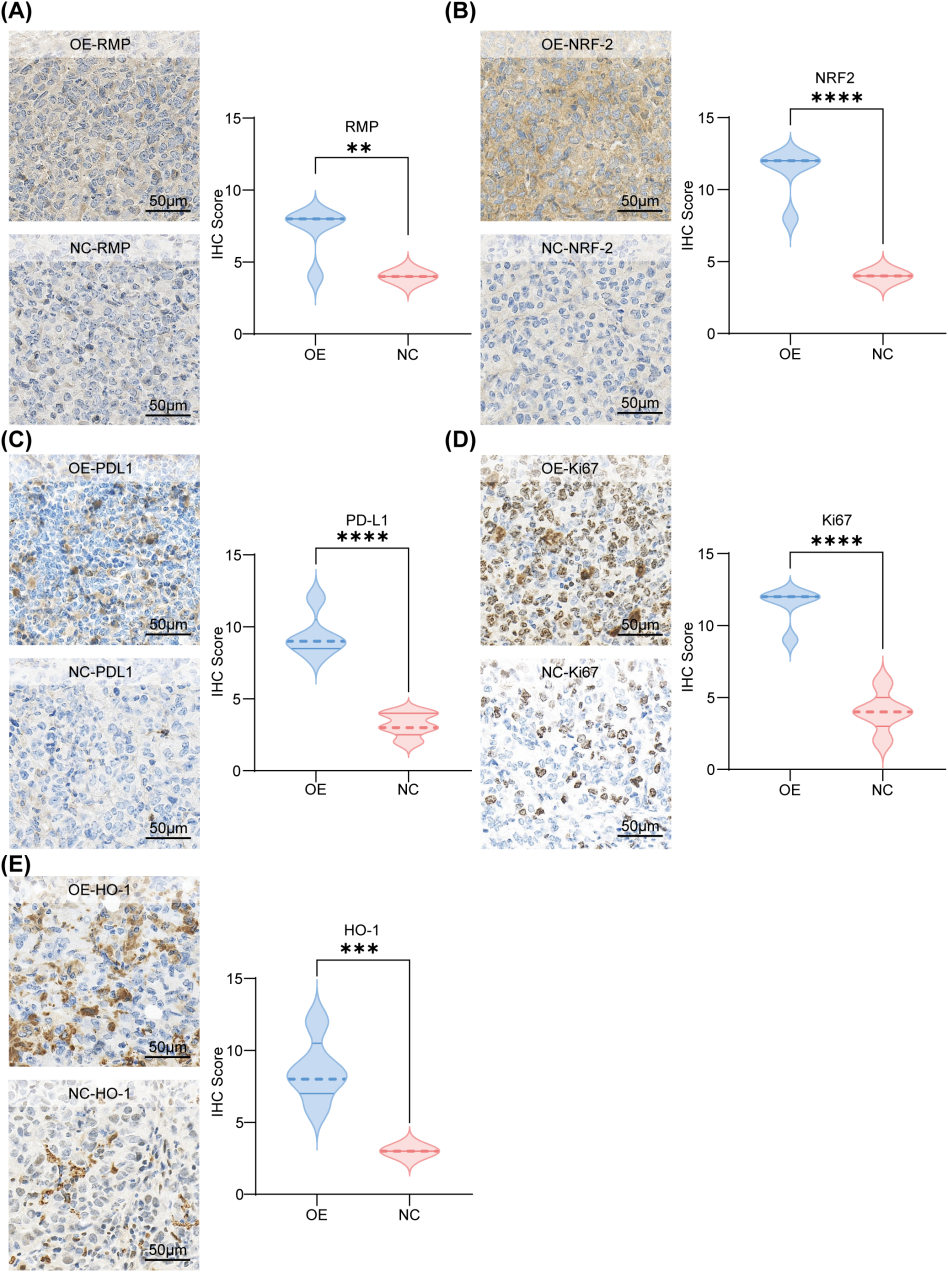
RMP−overexpressing tumors exhibit higher expression of RMP, NRF2, PD−L1, Ki−67, and HO−1 by immunohistochemistry. **(A)** Representative RMP immunohistochemical staining images (left) and IHC scores (right) of mouse tumor sections after the indicated treatments (scale bar: 50 μm; selected areas = 5). **(B)** Representative NRF2 immunohistochemical staining images (left) and IHC scores (right) (scale bar: 50 μm; selected areas = 5). **(C)** Representative PD-L1 immunohistochemical staining images (left) and IHC scores (right) (scale bar: 50 μm; selected areas = 5). **(D)** Representative Ki-67 immunohistochemical staining images (left) and IHC scores (right) (scale bar: 50 μm; selected areas = 5). **(E)** Representative HO-1 immunohistochemical staining images (left) and IHC scores (right) (scale bar: 50 μm; selected areas = 5). The data were presented as mean ± SD. and comparisons were performed with Student’s t-test; **p < 0.01; ***p < 0.001; ****p<0.0001.

Immunohistochemistry provided a molecular framework for understanding the growth advantage observed in OE tumors, which exhibited enhanced staining for RMP, thereby validating the genetic perturbation (**Figure 5A**). Similarly, NRF2 staining was elevated (**Figure 5B**), consistent with a redox-adaptive program. PD−L1 immunoreactivity was higher in OE tumors than in NC tumors (**Figure 5C**), consistent with the increased PD−L1 protein levels observed *in vitro* (**Figure 2D**) and supported by the elevated cell−surface PD−L1 detected by flow cytometry (**Supplementary Figures S1A, B**). The proliferative index, as measured by Ki-67, was elevated in OE tumors (**Figure 5D**), and heme oxygenase-1 (HO-1), a canonical target of NRF2 that mediates oxidative stress responses, was also increased (**Figure 5E**). The collective increase in RMP, NRF2, Ki−67, and HO−1, together with higher PD−L1 immunoreactivity, characterizes a tissue state defined by proliferation and oxidative−stress adaptation. This integrated phenotype connects our cellular findings with tumor behavior: the NRF2/HO−1 shift is consistent with enhanced redox adaptation that may support tumor growth, while higher PD−L1 immunoreactivity suggests potential engagement of the PD−1/PD−L1 axis, which we test functionally by PD−1 blockade in the next section. The tissue data indicate that OE tumors display a redox−adapted and proliferative state, and the increased checkpoint−related signals provide a rationale to evaluate PD−1 blockade responsiveness in the subsequent experiments. This dual characteristic underpins the rationale for exploring PD-1 blockade therapy in the subsequent section. The *a priori* expectation is that pathway engagement, a necessary condition for therapeutic response, is present. However, it is anticipated that additional resistance mechanisms associated with NRF2 and HO-1 may limit the extent of therapeutic benefit.

### 2.4 PD−1 blockade induces regression in both cohorts but proportionally less inhibition in the RMP/NRF2−high background

**Figure 6:**
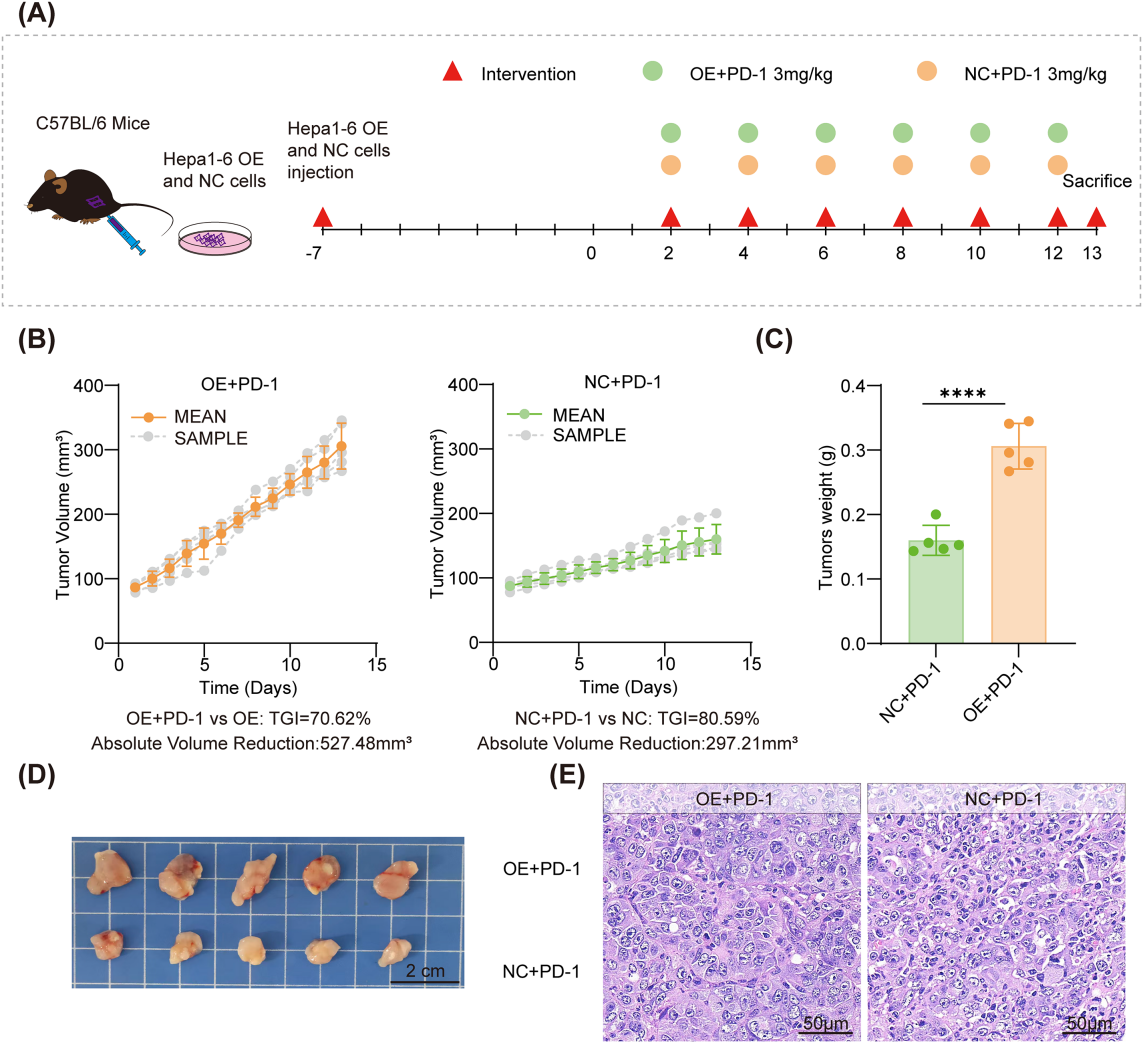
PD−1 blockade induces tumor regression in both cohorts; the RMP−overexpressing cohort shows reduced percent inhibition and remains larger at endpoint. **(A)** Study design: Mice were inoculated on day 0; anti−PD−1 (3 mg/kg, intraperitoneal) was administered every other day for six doses once tumors reached 50–100 mm³. **(B)** Tumor-growth curves showing individual mice and group mean ± SD.**(C)** Endpoint tumor weights, higher in the OE+PD-1 group than in the NC+PD-1 group. **(D)** Representative images of excised tumors (scale bars: 2 cm). **(E)** Representative H&E staining of tumor sections (scale bars, 50 μm). Data are mean ± SD; biological replicates are individual mice (n=5). Tumor weights were compared with Student’s t-test, ****p<0.0001.

To determine whether the composite RMP, NRF2, and PD−L1 state influences the magnitude of response to PD−1 blockade, we treated tumor−bearing mice with an anti−PD−1 antibody administered every other day for six doses at 3 mg/kg, beginning once tumors reached 50–100 mm³ (n=5 per genotype). (**Figure 6A**) Throughout the treatment process, the mice exhibited no significant reduction in body weight, and the structural integrity of their major organs remained intact (**Supplementary Figures S7A, B**). Both the NC and OE cohorts demonstrated tumor regression during therapy, as evidenced by decreases in tumor volume and reductions in endpoint tumor weight compared to their respective untreated controls (see **Figures 6B–E** in relation to the growth profiles in **Figures 4D, E**). Our findings also suggest that the absolute reduction in tumor size may be more pronounced in the OE group, even though this group presented with larger tumors at baseline. This observation partially indicates that the RMP-NRF2-PD-L1 axis plays a significant role in the response to PD-1 blockade in HCC and may be associated with the immune environment, and further analysis is warranted. However, when normalizing the treatment effect within each genotype using tumor−weight inhibition, the inhibition rates were 64.34% in NC tumors and 63.30% in OE tumors, supporting a comparable—but not enhanced—proportional response in the RMP/NRF2−high context. This finding aligns with the hypothesis that the NRF2-skewed background limits the efficacy of anti-PD-1 monotherapy. This relationship is clearly reflected in the endpoint weights, where OE tumors under anti-PD-1 treatment (OE+PD-1) remain heavier than NC tumors under anti-PD-1 treatment (NC+PD-1) (**Figure 6C**), despite both cohorts showing a response to therapy.

### 2.5 Adaptive−resistance pattern after PD−1 blockade: persistent NRF2–HO−1/PD−L1 activity and a CD8−enriched, inflamed−but−suppressed infiltrate in OE tumors

**Figure 7:**
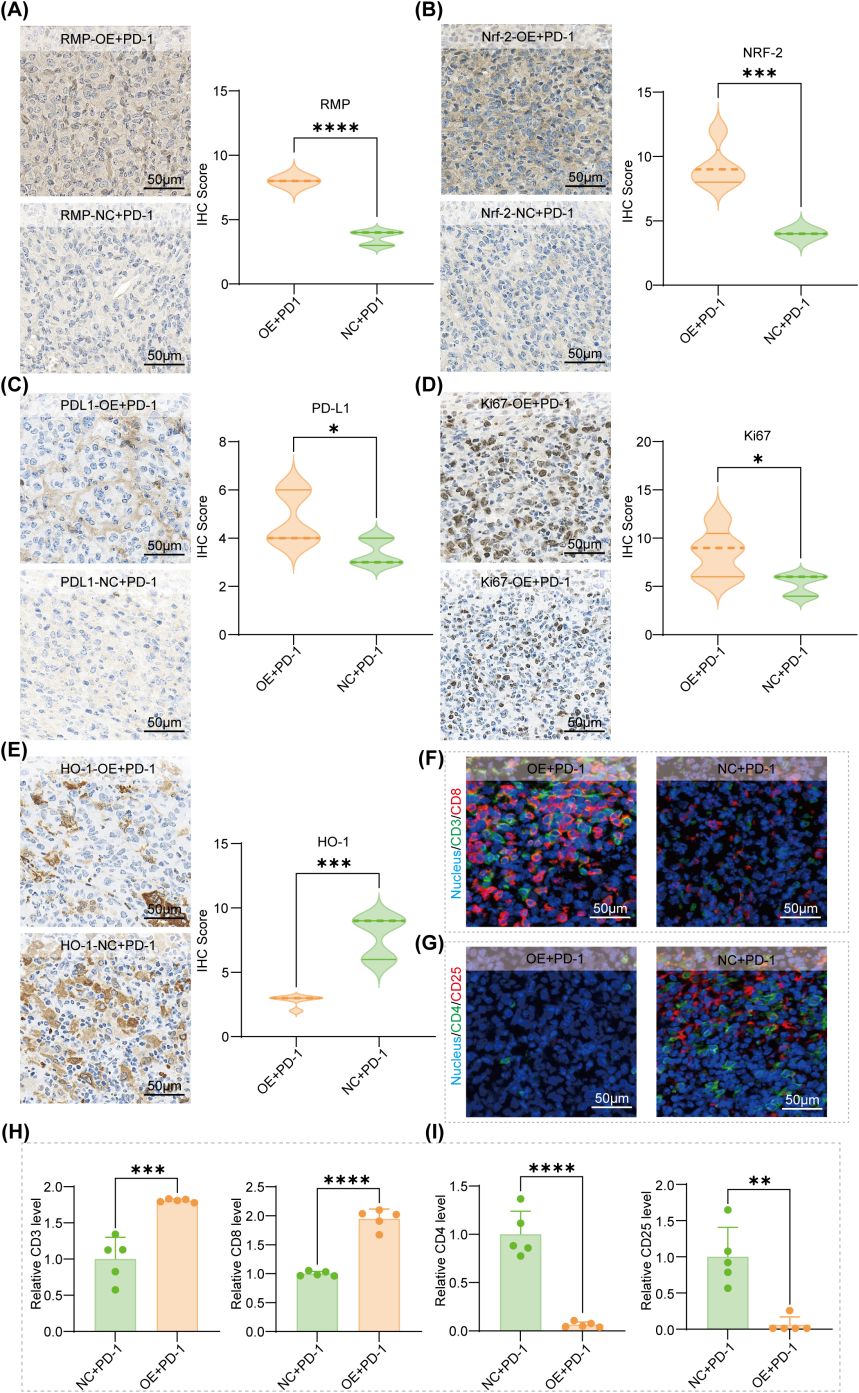
IHC and dual-immunofluorescence profiling in OE+PD-1 vs. NC+PD-1 tumors. **(A–E)** Representative IHC images (left) and IHC scores (right) for RMP **(A)**, NRF2 **(B)**, PD-L1 **(C)**, Ki-67 **(D)**, and HO-1 **(E)** in mouse tumor sections after the indicated treatments (scale bar: 50 μm; selected areas = 5 per sample). **(F)** Representative dual immunofluorescence images of CD3 (green) and CD8 (red) with nuclear counterstain (DAPI) in OE+PD-1 and NC+PD-1 tumors. **(G)** Representative dual immunofluorescence images of CD4 (green) and CD25 (red) with DAPI. **(H)** Quantification of relative fluorescence intensity (RFI) for CD3 and CD8 (scale bar: 50 μm; selected areas = 5). **(I)** Quantification of relative fluorescence intensity (RFI) for CD4 and CD25 (scale bar: 50 μm; selected areas = 5). The data were presented as mean ± SD (n=5) and comparisons were performed with Student’s t-test, *p < 0.05; **p < 0.01; ***p < 0.001; ****p < 0.0001.

At the conclusion of PD-1 therapy, we conducted a comprehensive profiling of tumor tissues to evaluate whether RMP overexpression continues to influence the tumor microenvironment under checkpoint blockade. Immunohistochemical analysis showed that the staining intensities of RMP and NRF2, as well as PD−L1 immunoreactivity, remained higher in OE+PD−1 tumors than in NC+PD−1 tumors (**Figures 7A–C**). These patterns indicate that the oxidative−stress and checkpoint−related signals associated with RMP remain comparatively higher under PD−1 therapy. Additionally, Ki-67 levels were higher in OE plus PD-1 tumors (**Figure 7D**), indicating a sustained proliferative drive, while heme oxygenase-1 levels also remained elevated (**Figure 7E**), suggesting that NRF2-dependent redox adaptation persists despite therapy. These findings are compatible with a growth−permissive, redox−adapted state that may contribute to a constrained response amplitude under PD−1 blockade.

Dual-color immunofluorescence provided valuable insights into the immune context. The signals for CD3 and CD8, which are indicative of overall T-cell presence and cytotoxic T-cell infiltration, were more pronounced in the OE+PD-1 group compared to the NC+PD-1 group. Conversely, the signals for CD4 and CD25, which serve as proxies for regulatory T-cell characteristics, were generally lower in the OE+PD-1 group. Quantitative data are presented as relative fluorescence intensities across five selected regions per sample (**Figures 7F–I**). To achieve a more precise cell-based assessment, we further quantified the densities of CD3^+^CD8^+^ and CD4^+^CD25^+^ double-positive cells. These measurements mirrored the directional changes identified through RFI analysis, as shown in **Supplementary Figures S8A, B**. Collectively, these post−therapy tissue data complement the macroscopic response profile and suggest that while PD−1 blockade elicits cytotoxic T−cell infiltration, the axis linking RMP, NRF2, and heme oxygenase−1 remains active and PD−L1 remains elevated, maintaining inhibitory pressure that likely explains the lower percent inhibition in OE compared with NC.

## 3 Discussion

Hepatocellular carcinoma (HCC) exhibits variable responsiveness to PD-1/PD-L1 blockade, and there is ongoing debate regarding the adequacy of using PD-L1 as a solitary predictive marker. Additionally, the influence of redox programs, particularly the KEAP1-NRF2 axis, on immune phenotypes—ranging from immune exclusion to an inflamed yet suppressed state—remains a subject of investigation (9, 36–39). In this context, the extent to which RMP integrates NRF2-driven redox adaptation with PD-L1-centered immune regulation in HCC has not been clearly elucidated (31, 40–42). To address this, we engineered hepatoma cells to overexpress RMP, demonstrating increased levels of NRF2 and PD-L1 proteins, along with enhanced clonogenic growth and migration capabilities. Mechanistically, although we did not directly quantify NFE2L2 transcription or determine the half-life of NRF2, our protein data align most closely with a post-translational stabilization model. In this model, RMP/URI1 interacts with KEAP1, thereby attenuating KEAP1-dependent ubiquitination and degradation of NRF2 (32). Supporting this concept, recent studies have increasingly emphasized druggable protein–protein interactions that regulate NRF2 stability and signaling pathways in cancer (43). Furthermore, downstream NRF2 signaling has been associated with the modulation of PD-L1 expression in hepatobiliary tumor models. Pharmacological inhibition of NRF2 has been shown to reduce PD-L1 levels, consistent with our Brusatol validation. However, additional regulatory mechanisms, such as PD-L1 protein turnover, cannot be ruled out (44). Because clonogenic assays integrate survival and proliferative capacity and scratch closure can be partially influenced by proliferation, we interpret the increased colony formation and wound closure in RMP-OE cells as a composite gain in tumor-cell fitness rather than a purely migration-specific effect.

*In vivo*, tumors overexpressing RMP exhibited accelerated growth and demonstrated a convergent tissue signature characterized by elevated levels of RMP, NRF2, PD-L1, and increased expression of Ki-67 and HO-1. This profile is indicative of a state adapted to proliferative and oxidative stress, with high expression of checkpoint ligands. Under anti-PD-1 therapy, tumor regression was observed in both genetic backgrounds. However, due to the initially larger size of tumors in the overexpression cohort, the absolute reduction in tumor size was potentially greater in this group. Nevertheless, when normalized within each genotype, the overexpression cohort showed a lower percentage of inhibition, suggesting a limited response amplitude in the RMP/NRF2-high context. Post-therapy analysis of tissues from the overexpression cohort revealed sustained elevated levels of RMP, NRF2, HO-1, and PD-L1. Dual-color immunofluorescence analysis indicated increased CD3/CD8 signals alongside reduced CD4/CD25 signals, reflecting an immune contexture that is inflamed yet functionally restrained, described as “hot yet suppressed”. The concomitant increase in CD8-associated signals with persistent PD-L1 expression after PD-1 blockade is compatible with an IFN-γ–linked adaptive resistance program, supporting the “hot-yet-suppressed” framework and offering a mechanistic rationale for the reduced proportional inhibition observed in the RMP/NRF2-high background. Together, these findings support a model in which PD−1 blockade brings cytotoxic T−cell infiltration, but the axis linking RMP, NRF2, and HO−1 remains active and PD−L1 remains elevated, sustaining inhibitory pressure and providing a mechanistic explanation for the lower relative inhibition in the overexpression group compared with controls. Building on this, our work contributes a composite RMP/NRF2/PD−L1 signature, that reframes the controversy toward an adaptive−resistance paradigm and offers biomarker implications and provides a mechanistic rationale to motivate future testing of redox−modulating co−treatments with PD−1 blockade in HCC.

Looking forward, our findings suggest that context−aware biomarkers integrating redox status with checkpoint features may help refine stratification in HCC; however, the therapeutic benefit of redox−pathway co−targeting with PD−1 blockade remains to be demonstrated (45). From a translational perspective, the direct pharmacological inhibition of RMP/URI1 remains challenging; thus, therapeutically modulating the downstream NRF2 pathway may offer a more viable short-term strategy (46). Notably, a recent small-molecule screening identified the FDA-approved antifolate pyrimethamine as an NRF2 inhibitor, which facilitates NRF2 degradation and suppresses NRF2-high phenotypes *in vivo* (47). Emerging evidence further suggests that NRF2 signaling can influence antitumor immunity. For instance, the KEAP1–NRF2 axis in CD8^+^ T cells induces terminal exhaustion via the prostacyclin receptor PTGIR, highlighting potentially druggable targets downstream of NRF2 (48). Alongside the recent development of orally bioavailable HO-1 inhibitors that reprogram tumor-associated macrophage functions and enhance CD8^+^ T-cell infiltration (30), these developments provide feasible pharmacological tools to interrogate NRF2/HO−1 modulation in preclinical models, and support the testability of redox−pathway co−targeting alongside PD−1 blockade.

Our findings identify a candidate mechanistic signature (RMP/NRF2/PD−L1 with HO−1) that may inform biomarker development and generate testable hypotheses for future combination studies; however, clinical or *in vivo* therapeutic optimization was not directly evaluated here. We recognize the limitations of the present study, including the reliance on a single syngeneic model with a modest sample size for *in vivo* validation. Subsequent investigations should aim to validate this signature in orthotopic and other immunocompetent models, integrate a broader range of immune cell populations, and explore combinations of NRF2/HO-1 inhibition with PD-1 blockade, among other strategies. In conclusion, this study delineate how RMP connects NRF2−driven redox adaptation with PD−L1–mediated adaptive resistance under PD−1 therapy: PD−1 blockade increases cytotoxic T−cell infiltration, yet persistent RMP-NRF2-HO−1 activity and sustained PD−L1 expression maintain inhibitory pressure, explaining the lower relative inhibition in the overexpression setting and providing a mechanistic, composite framework to inform biomarker development and support future evaluation of rationally designed combination regimens in HCC.

## Data Availability

The original contributions presented in the study are included in the article/**Supplementary Material**. Further inquiries can be directed to the corresponding author.
