# Supplementary material for: Hot−yet−suppressed under PD−1 blockade: an RMP–NRF2–PD−L1 axis associated with a reduced proportional response in hepatocellular carcinoma
Source: Front Immunol. 2026 Feb 5;17:1737569. doi: 10.3389/fimmu.2026.1737569 (PMC12916707; doi:10.3389/fimmu.2026.1737569)
Supplement: Supplementary file 1 [file DataSheet1.docx]

Supplementary Material

# Material and method

## Cell Culture and Establishment of RMP-Overexpressing Cell Line

In this study, mouse hepatocellular carcinoma (Hepa1-6) and Hep3B cells were obtained from the Shanghai Cell Bank of the Chinese Academy of Sciences. The cells were cultured in DMEM supplemented with 10% fetal bovine serum (FBS) under a humidified atmosphere at 37°C with 5% CO₂. The gene sequence for overexpression and the RMP-overexpressing lentiviral vector were constructed and packaged by OBIO Technology (Shanghai, China). For lentiviral infection, the overexpression (OE) group mixture consisted of 2.54 × 10⁸ transducing units (TUs) of lentivirus, 2.5 μL of polybrene infection enhancer, and 500 μL of complete medium. The negative control (NC) group mixture contained 6.97 × 10⁸ TUs of lentivirus, 2.5 μL of polybrene, and 500 μL of complete medium. These mixtures were used to infect Hepa1-6 cells seeded in 24-well plates at approximately 15% confluence. Specifically, 500 μL of the virus-containing medium was added to the cells. After 48 hours of incubation, the medium was replaced with fresh complete medium, and the cells were further cultured for 24 hours. Subsequently, the cells were selected with complete medium containing 10 μg/mL puromycin (puro) for 48 hours.

## Western blotting (WB)

Following treatment, cells were lysed using RIPA buffer (Beyotime, Shanghai, China) supplemented with a protease inhibitor cocktail to minimize protein degradation. Protein concentrations were determined through bicinchoninic acid (BCA) quantification (Beyotime, Shanghai, China). The protein components from the lysates were separated by 10% sodium dodecyl sulfate-polyacrylamide gel electrophoresis (SDS-PAGE) and subsequently electrophoretically transferred onto polyvinylidene difluoride (PVDF) membranes (Millipore, Burlington, MA, USA). Primary antibodies employed in this work included those against GAPDH (6000-1-Ig, dilute 1:5000, Proteintech, Wuhan, Hubei, China), RMP (11277-1-AP,dilute 1:1000, Proteintech, Wuhan, Hubei, China), NRF-2 (80593-1-RR, dilute 1:1000, Proteintech, Wuhan, Hubei, China) and PD-L1/CD247 (66248-1-Ig, dilute 1:5000, Proteintech, Wuhan, Hubei, China).

## Colony Formation Assay

Hepa1-6 and Hep3B cells stably overexpressing the target gene (OE) and negative control (NC) cells were seeded in 6-well plates at a density of 800 cells per well. After 10 days of incubation, the cells were washed gently 2-3 times with phosphate-buffered saline (PBS) to avoid dislodging the adherent colonies. Subsequently, the colonies were fixed with 4% paraformaldehyde (1 mL/well) for 15–20 minutes at room temperature. After fixation, the cells were washed 2-3 times with PBS and then stained with 0.1% crystal violet (1 mL/well) for 15–30 minutes. Excess dye was removed by thoroughly rinsing the wells with purified water. The plates were air-dried, and colonies were imaged.

## Cell migration

Hepa1-6 NC and OE cells were seeded in 6-well plates at a density of 4×10⁵ cells per well and allowed to adhere overnight. After complete attachment, the cells were gently washed with PBS. A scratch was created in each well using a sterile tip, followed by two washes with PBS. Images were captured at the 0 h time point. Then, 1 mL of complete medium was added to each well, and the cells were further incubated for 24 h before subsequent imaging.

## Animal experiments and model construction

Every mouse was acquired from Xi’an Jiaotong University’s Laboratory Animal Center. The animals were kept in standard housing with normal light/dark cycles, standard feed, and no specific pathogens. Every animal experiment was carried out in compliance with Institution Guidelines and authorized by Xi'an Jiaotong University's Medical Ethics Committee (permission number: 2022-370). Hepa1-6 overexpression (OE) and negative control (NC) hepatocellular carcinoma tumors were established in the right flank of 4-week-old female C57/BJ mice, with 5×10⁶ cells inoculated per mouse.

## *In vivo* Antitumor Experiments

Hepa1-6 overexpression (OE) and negative control (NC) hepatocellular carcinoma tumors were established in the right flank of 4-week-old female C57/BJ mice, with 5×10⁶ cells inoculated per mouse. Administration was initiated when the tumor volume reached 50-100 mm³ (n=5 per group). Each mouse was administered at a dose of 3 mg/kg in a volume of 100 μL, once every other day, for a total of 6 administrations.

Throughout the administration period, the body weight of the mice and tumor volume (calculated using the formula: 1/2 × length × width²) were monitored. Anesthesia was administered through intraperitoneal injection of tribromoethanol (TBE，1.25%) at a dosage of 0.2 mL/10 g body weight. For euthanasia, cervical dislocation was performed on all mice. Death was confirmed upon cessation of respiratory movement, absence of abdominal breathing motion, and no withdrawal reflexes observed upon gentle pinching of the limbs.Then, immediately afterward, each mouse was necropsied on an ice surface to acquire samples of tumor and the weight of the excised tumors was recorded. The tumors were then fixed in 4% paraformaldehyde, dehydrated, and embedded in paraffin. Subsequently, 5 μm-thick sections were prepared for hematoxylin-eosin (H&E) staining or immunohistochemical (IHC) staining.

## Immunohistochemistry score

For the evaluation of immunostaining intensity (I), a numeric score ranging from 0 to 3 was employed, reflecting the intensity as follows: 0 denoting no staining, 1 indicating weak staining, 2 signifying moderate staining, and 3 representing intense staining. In the case of assessing immunostaining area (A), a numeric score ranging from 1 to 4 was utilized, corresponding to the following categories: 1 representing a positive area of <25%, 2 indicating a positive area between 25% and 50%, 3 reflecting a positive area spanning 51% to 80%, and 4 denoting a positive area exceeding 80%. Using an Excel spreadsheet, the mean score was obtained by multiplying the intensity score (I) by the percentage of the positive area, and the results were added together (total score: I×A).

## Immunofluorescence staining

For tissues immunofluorescence, tumor sections were stained with primary antibodies then with the corresponding Alexa Fluor-488 or cy3-conjugated secondary antibody. Nextly, all slides stained with antibodies were mounted with Vectashield Mounting Medium containing 4’-6-diamidino-2-phenylindole (DAPI). The primary antibodies were used: CD3, CD8(Servicebio, China;1:10000), CD4(Abcam, USA;1:10000), CD25(CST, USA;1:2000). Images were obtained by 3DHISTECH slide scanning system (3DHISTECH, Hungary).

## TCGA data processing and immune infiltration analysis

We downloaded RNA-seq data (STAR-counts) and corresponding clinical information for liver hepatocellular carcinoma (LIHC) from The Cancer Genome Atlas (TCGA). Raw read counts were converted to transcripts per million (TPM) values using gene length annotation, followed by log2(TPM + 1) transformation for normalization. After retaining samples with matched RNA-seq and clinical information, a total of 371 LIHC samples were included for downstream analyses.Gene–gene correlations were evaluated using Spearman’s correlation, and visualization was performed with the ggstatsplot R package. Immune cell infiltration was estimated using the immunedeconv R package, which integrates TIMER, xCell, MCP-counter, CIBERSORT, EPIC, and quanTIseq. Unless otherwise specified, immune infiltration was primarily assessed using the CIBERSORT algorithm.Statistical analyses were conducted in R (v4.0.3).

## Flow cytometry

Tumor cells were dissociated into single-cell suspensions and washed with staining buffer(D-PBS+1%FBS). Cells (1 × 10^6^ per sample) were incubated with Brilliant Violet 421™ anti-mouse CD274(124315,BioLegend, San Diego, CA, USA) for 30 min at 4℃ in the dark. After washing, cells were stained with BD Horizon™ Fixable Viability Stain 700 (564997,BD Horizon™,Becton, Dickinson and Company, Franklin Lakes, NJ, USA) in staining buffer for 10–15 min (protected from light), and the free dye was quenched by washing with staining buffer.Samples were acquired on a flow cytometer (Beckman Coulter CytoFLEX LX,Brea, CA, USA) and analyzed using FlowJo. Singlets were gated based on FSC-A/FSC-H, and dead cells were excluded as FVS700 high events. PD-L1 expression was reported as percentage of PD-L1 positive cells.

## Oxidative Stress Model

Hepa1-6 overexpressing (OE) cells were seeded in 6-well plates at a density of 4×10⁵ cells per well. After overnight adhesion, the cells were treated with 50 μM hydrogen peroxide solution. Following a 4-hour incubation, total proteins were extracted, and the expression changes of NRF-2 and PD-L1 proteins were detected via Western blot (WB) assay.

## Statistical Analysis

The data were presented as mean ± standard deviation (SD), and statistical analyses were conducted using two-tailed Student’s t-test. Correlation analysis was conducted using the Spearman test. Sample size (n) for each statistical analysis was 3 or 5. Statistical analyses were performed using GraphPad Prism 10.1.2. A significance level of P < 0.05 was considered statistically significant.

# Supplementary Figures


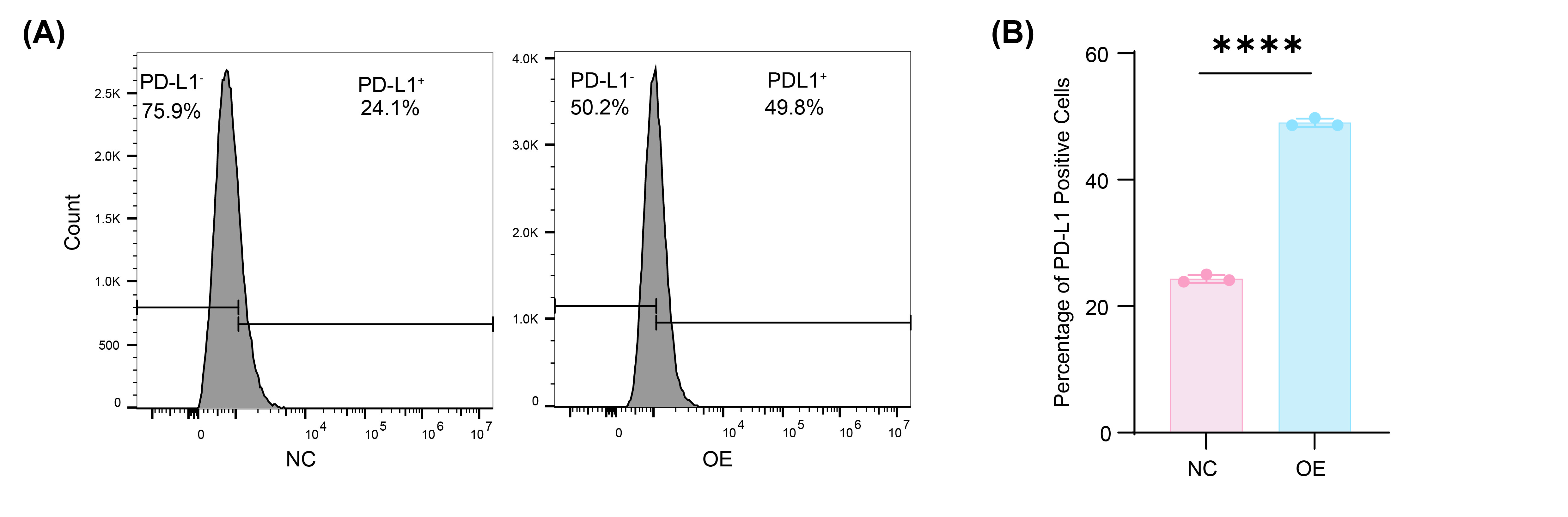


**Supplementary Figure S1**. RMP overexpression increases PD-L1 expression on the cell surface of Hepa1-6 cells. **(A)** Representative flow cytometry histograms showing cell surface PD-L1 expression in NC or OE Hepa1-6 cells. **(B)** Quantification of the percentage of PD-L1–positive cells in NC and OE groups. Data represent mean ± SD from independent experiments (n = 3). Statistical significance was assessed by unpaired two-tailed Student’s t-test; ****p < 0.0001.


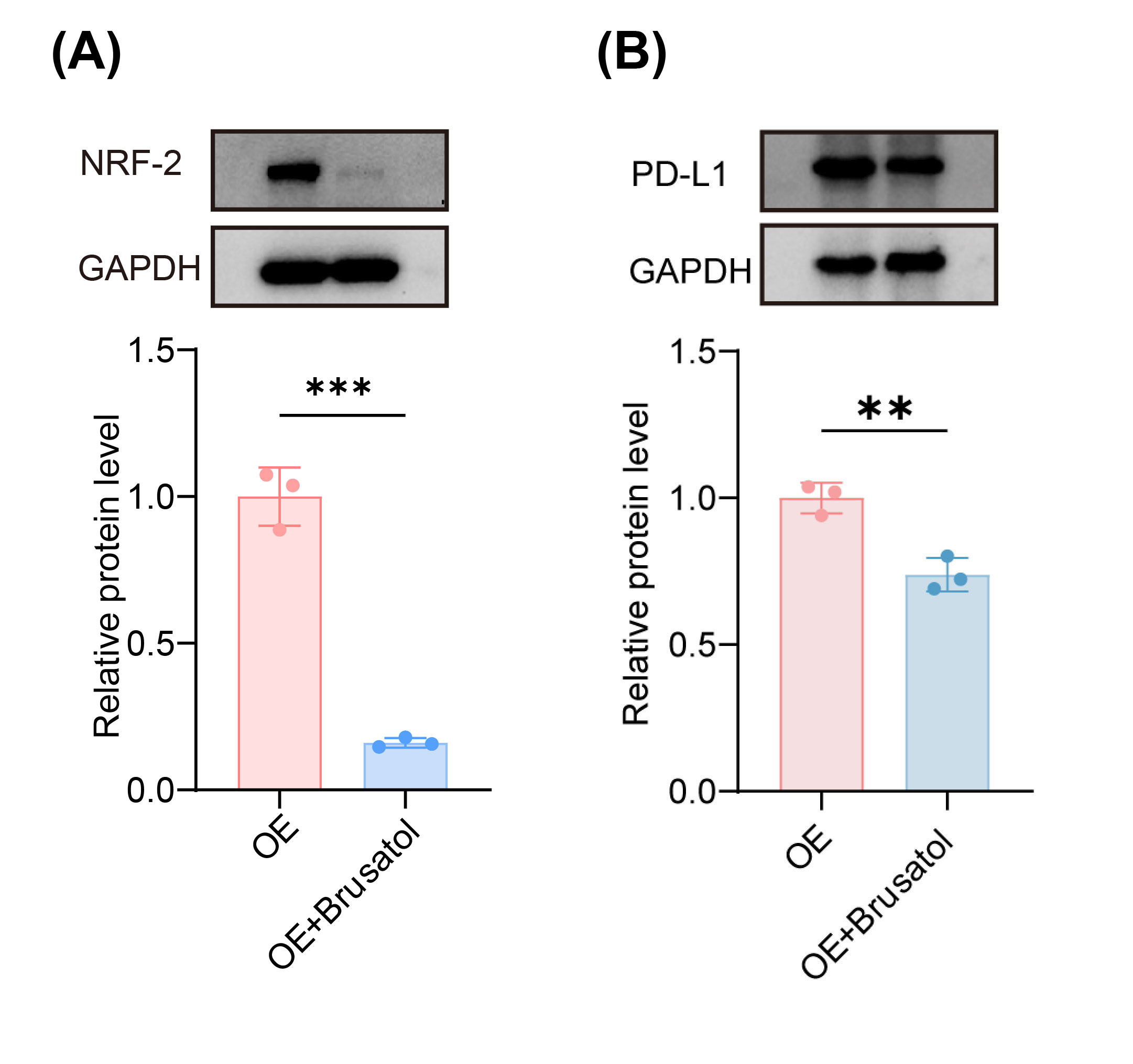


**Supplementary Figure S2**. Brusatol-mediated NRF2 inhibition downregulates PD-L1 expression in Hepa1-6 (OE) cells. **(A)** Representative blots and densitometric quantification of NRF2. **(B)** Representative blots and densitometric quantification of PD-L1. Data are presented as mean ± SD, n=3 independent experiments and comparisons were performed with Student’s t-test; **p<0.01, ***p<0.001.


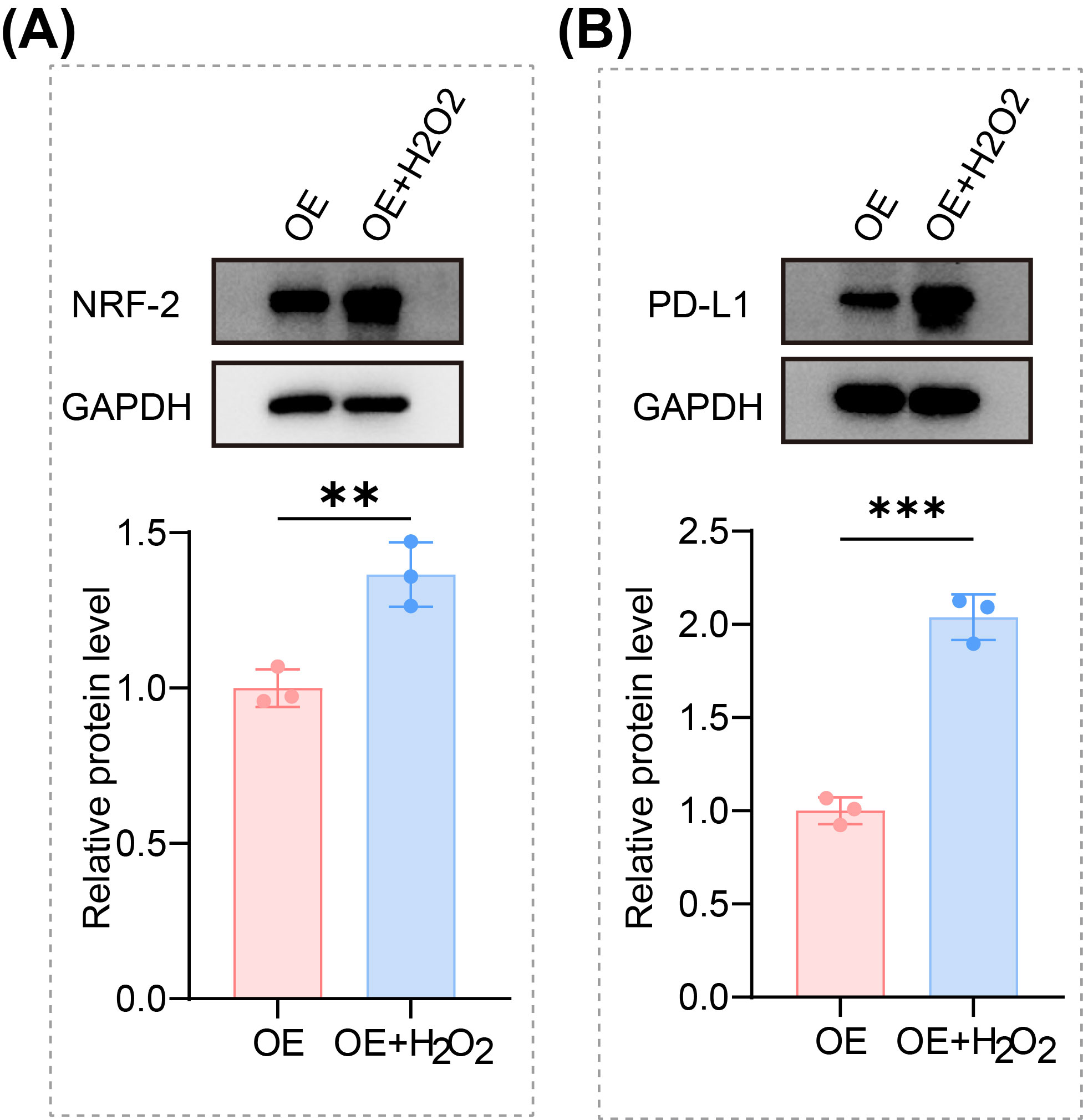


**Supplementary Figure S3.** Oxidative stress upregulates NRF-2 and PD-L1 in RMP overexpressed Hepa1-6 overexpressing cells. **(A)** Representative blots and densitometric quantification of NRF2. **(B)** Representative blots and densitometric quantification of PD-L1. Data are presented as mean ± SD, n=3 independent experiments and comparisons were performed with Student’s t-test; **p<0.01, ***p<0.001.


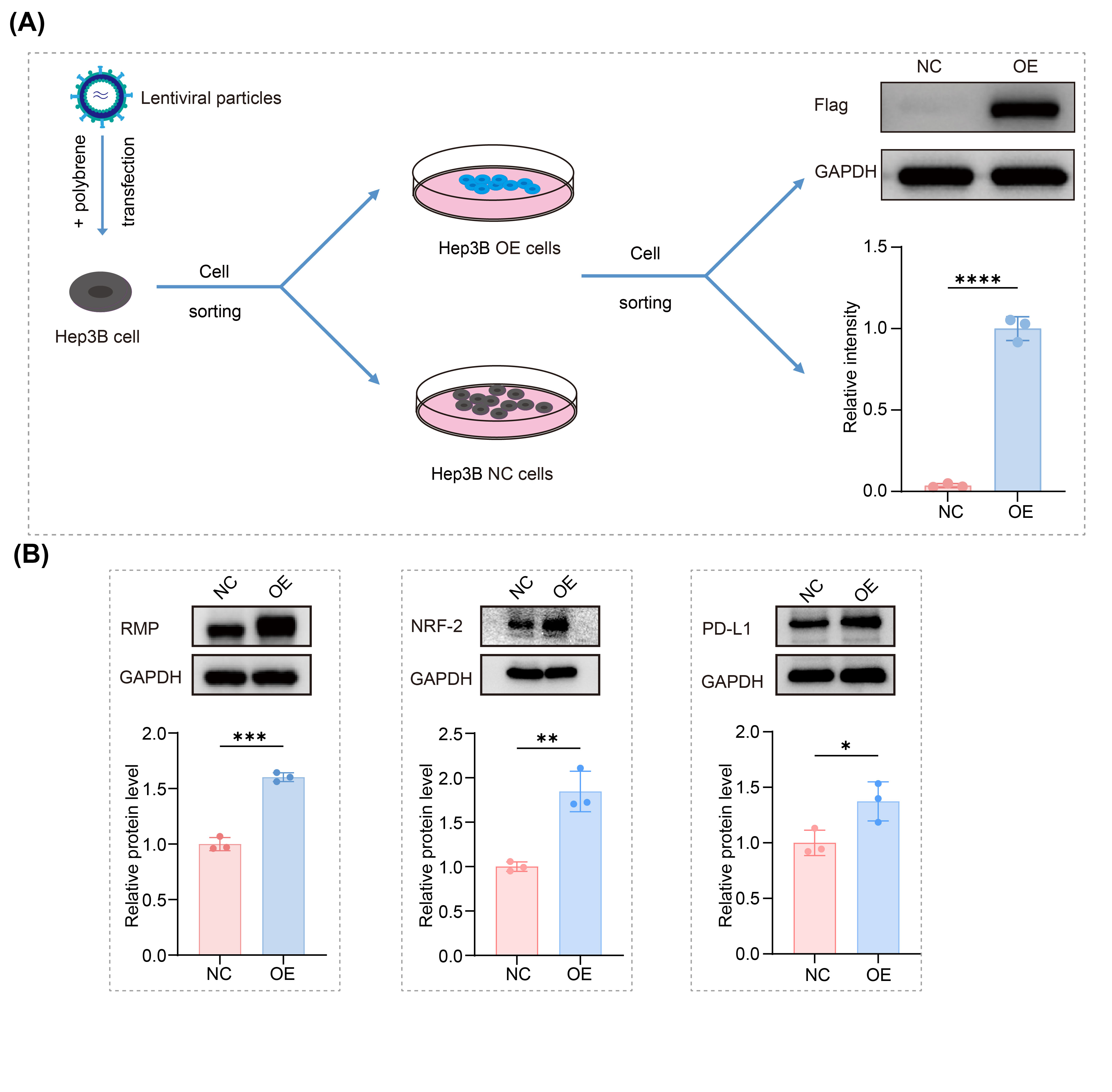


**Supplementary Figure S4**. RMP overexpression increases NRF2 and PD‑L1 protein levels in Hep3B cells. (A) Schematic of lentiviral transduction (with polybrene) and sorting to establish RMP-overexpression (OE) and negative control (NC) Hep3B cells. Representative Western blot validating RMP overexpression using a Flag tag. (B) Representative blots and densitometric quantification of RMP, NRF2, and PD-L1. Data are presented as mean ± SD, n=3 independent experiments and comparisons were performed with Student’s t-test; *p<0.05, **p<0.01, ***p<0.001, ****p<0.0001.


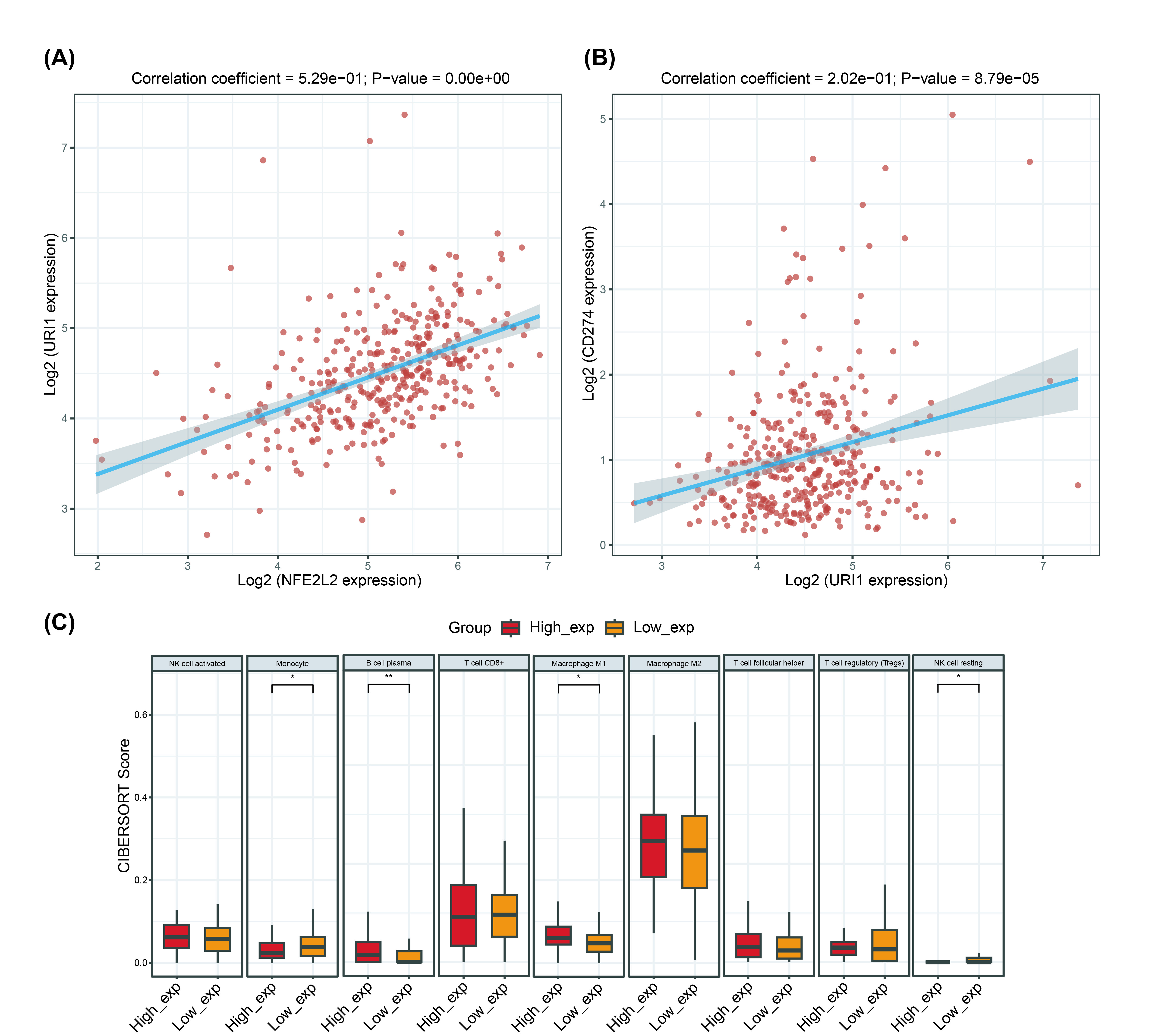


**Supplementary Figure S5.** TCGA hepatocellular carcinoma cohort reveals coordinated expression patterns of NFE2L2/URI1 and URI1/PD-L1 and their association with immune infiltration. **(A)** The correlation between NFE2L2(NRF2) and URI1(RMP) expression levels in the TCGA hepatocellular carcinoma (HCC) cohort. **(B)** The correlation between URI1 and CD274 (PD-L1) expression levels in the TCGA HCC cohort. **(C)** Comparison of CIBERSORT-estimated immune cell infiltration between RMP and PD-L1 double-high (High_exp) and double-low (Low_exp) expression groups in TCGA HCC samples; the distributions of immune infiltration scores across multiple immune cell subsets are shown for each group. The significance of two groups of samples is evaluated by the Wilcoxon rank-sum test. (*p < 0.05, **p < 0.01)


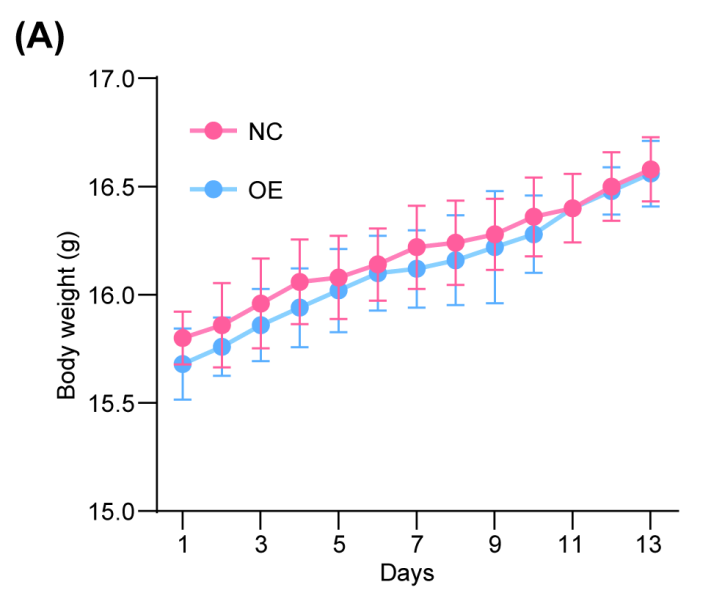


**Supplementary Figure S6.** Body-weight trajectories of NC and OE cohorts.


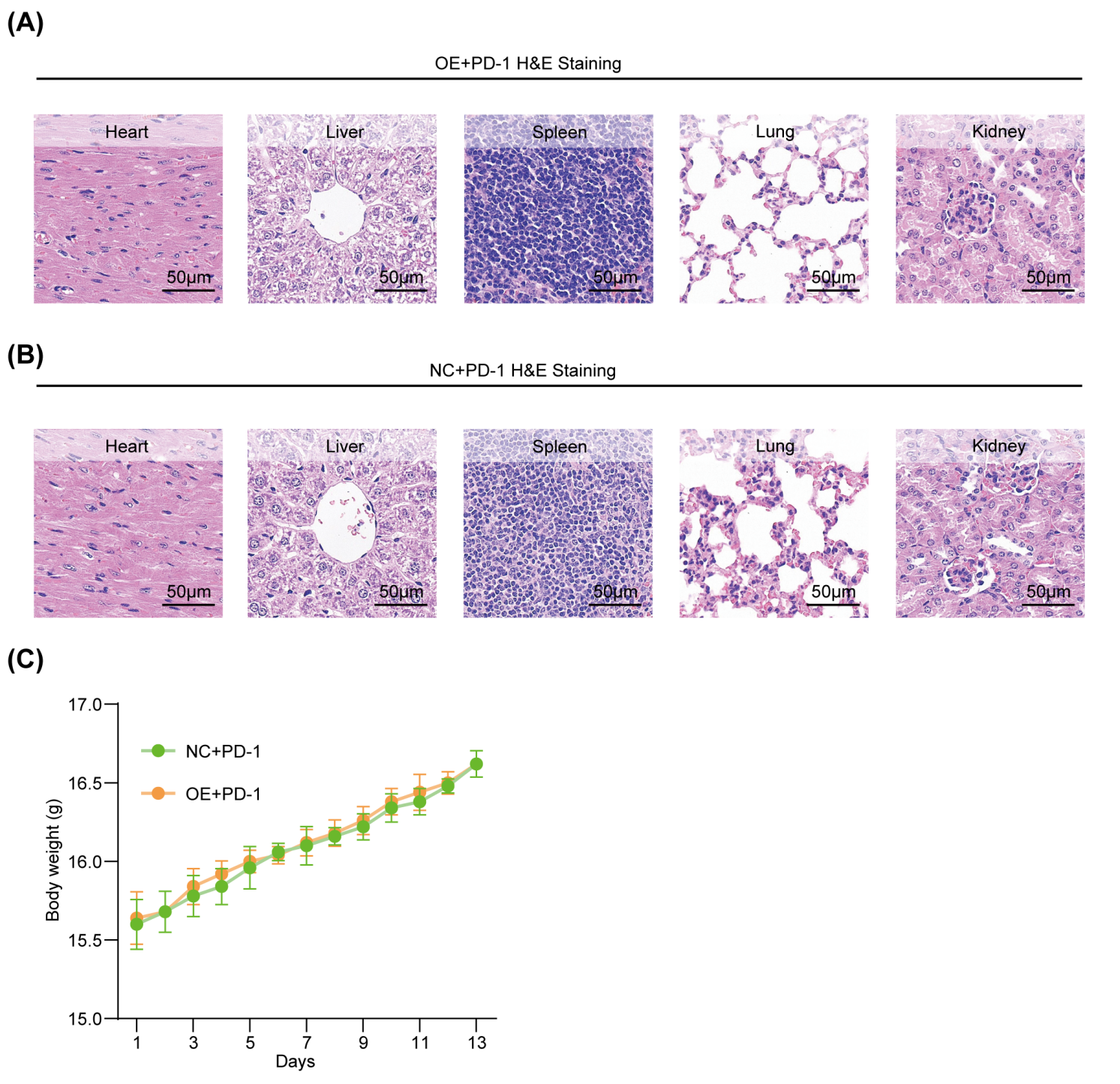
**Supplementary Figure S7**. H&E evaluation of major organs and body-weight monitoring under PD-1 therapy. **(A)** Representative H&E staining of heart, liver, spleen, lung, and kidney from the OE+PD-1 group (scale bars, 50 µm). **(B)** Representative H&E staining of the same organs from the NC+PD-1 group (scale bars, 50 µm). No overt histopathological abnormalities are observed in either group.
**(C)** Body-weight curves of mice during treatment (mean ± SD, groups as indicated).


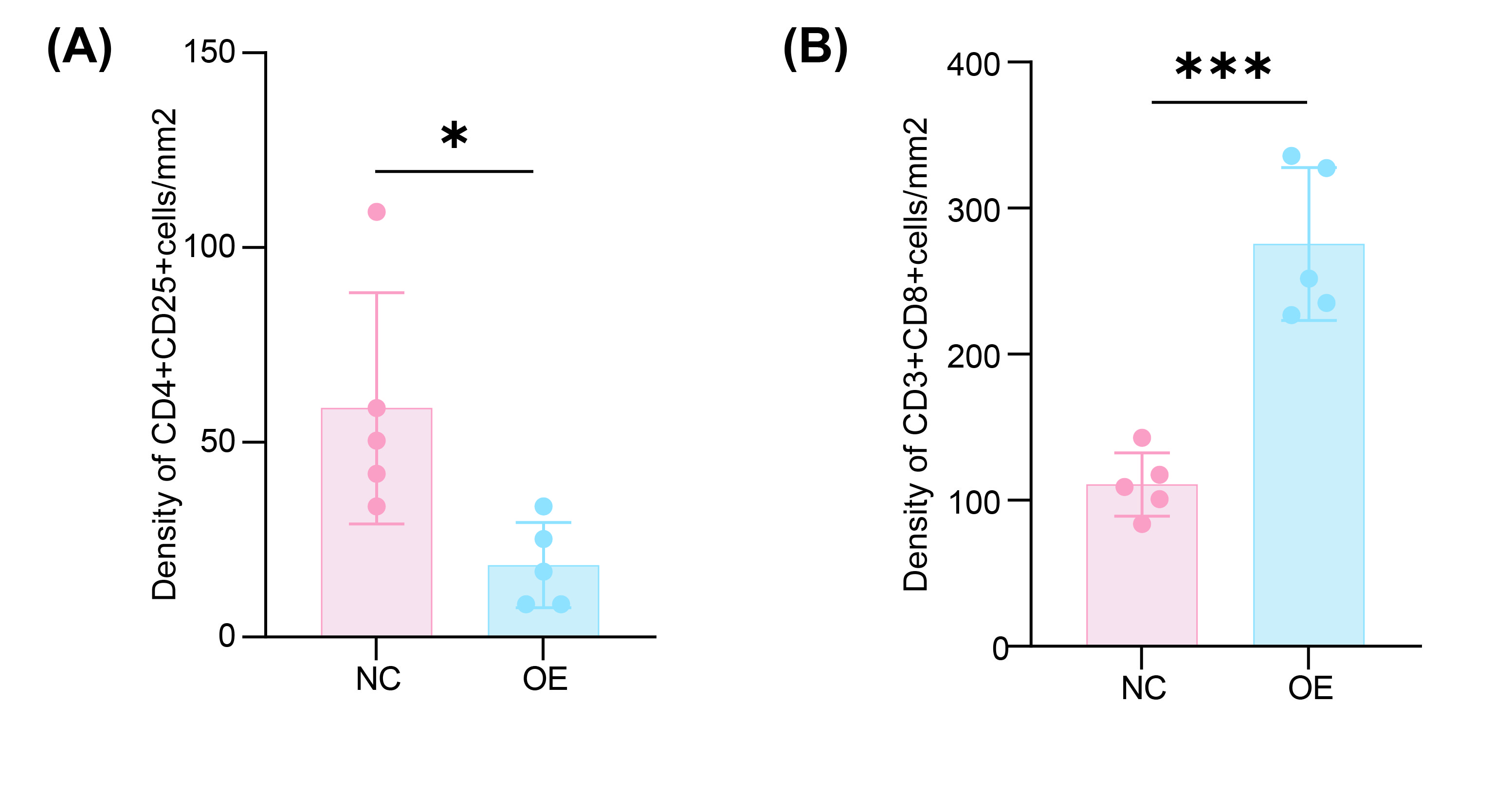


**Supplementary Figure S8.** PD-1 treatment alters tumor-infiltrating T cell densities in OE+PD-1 and NC+PD-1 groups. **(A)**Quantification of density of CD4⁺CD25⁺ cells (cells/mm²) (selected areas = 5). **(B)** Quantification of density of CD3⁺CD8⁺ cells (cells/mm²) (selected areas = 5). The data were presented as mean ±SD. and comparisons were performed with Student’s t-test, *p < 0.05; ***p < 0.001.
